# Impact of educational intervention regarding COVID-19 on knowledge, attitude, and practice of students before dental school re-opening

**DOI:** 10.1186/s12903-023-02845-y

**Published:** 2023-03-18

**Authors:** Arghavan Etebarian, Somayeh Khoramian Tusi, Zahra Momeni, Kimia Hejazi

**Affiliations:** 1 Department of Oral and Maxillofacial Pathology, School of Dentistry, Alborz University of Medical Sciences, Katouizadeh Sq., Golshahr Boulevard, Karaj, 3198684868 Iran; 2 Department of Pediatric Dentistry, School of Dentistry, Alborz University of Medical Sciences, Karaj, Iran; 3 Department of Community Oral Health, School of Dentistry, Alborz University of Medical Sciences, Karaj, Iran; 4 Student Research Committee, School of Dentistry, Alborz University of Medical Sciences, Karaj, Iran

**Keywords:** Education, Knowledge, Attitude, Practice management, COVID-19, Dental, University student

## Abstract

**Background and Aim:**

Lack of knowledge on COVID-19 among people and healthcare staff significantly impacts late management and its rapid transmission. Dental students must be aware of the exact preventive instructions due to their close contact with patients and clinical work in the dental setting during the pandemic. This interventional study aimed to investigate the effectiveness of designed educational content regarding COVID-19 on Iranian dental students’ knowledge, attitudes, and practices at Alborz University of medical sciences.

**Materials and methods:**

A total of 88 dental students were investigated in this study. Educational content was designed based on the clinical guidelines regarding COVID-19 in dentistry. The content was presented to dental students as a three-hour webinar. In addition, the online, validated questionnaire was obtained from the students as pre, post, and follow-up surveys.

**Results:**

A statistically significant difference between students’ knowledge, attitudes, and practices were obtained before and after the intervention and the follow-up survey. 48.58% improvement in knowledge score, 6.37% in attitudes, and 17% in practice scores were observed.

**Conclusion:**

Although this educational intervention significantly improved the knowledge, attitude, and practice of dental students, additional education and clinical training are mandatory for effective and safe dental practice management in future crises.

**Supplementary Information:**

The online version contains supplementary material available at 10.1186/s12903-023-02845-y.

## Introduction

Coronavirus disease 2019 (COVID-19) is contagious viral infection caused by the SARS-CoV-2 virus [1]. Coronavirus was first discovered in Wuhan, Hubei province, China, in December 2019, and on 11th March 2020, the WHO declared COVID-19 as a new pandemic [2]. Although most people infected with COVID-19 experience mild to moderate respiratory illness and recover without special treatment, it might develop and appear to be a severe disease leading to death [3]. Usually, older people and patients with systemic diseases such as cardiovascular disease, diabetes, chronic respiratory disease, or cancer are more likely to develop serious illnesses [4]. The main symptoms of COVID-19 are fever, cough, tiredness, and loss of taste or smell [5]. Nevertheless, less commonly, sore throat, headache, aches, pains, diarrhea, a rash on the skin, discoloration of fingers or toes, and red or irritated eyes may be seen in the patients [6]. Droplets and contact with contaminated surfaces remained the most frequent modes of transmission in COVID-19 [7, 8], and dental settings are high-risk environments due to producing aerosols and droplets [9, 10]. Education is one of the important measures that can help prevent and control infectious diseases in public, including health care staff [11]. Kaim and colleagues have studied the impact of a brief educational intervention through an online video among the Israeli public, and the results showed a significant increase in perceived knowledge [12]. So far, numerous cross-sectional studies have been done to assess knowledge, attitude, and practice regarding COVID-19 in Iran [13–16] and worldwide in different populations [4, 9, 17–20]. To date, there is no interventional study undertaken among dental students in Iran. Earlier in the Alborz dental school, a cross-sectional related survey indicated the need for educational intervention in this field [21]. The main goal of the present study was to investigate the impact of an educational intervention through an online, validated questionnaire on the knowledge, attitudes, and practices of Alborz dental students in Iran.

## Methods and materials

### Study design and participants

Considering the importance of education among dental students in clinical settings, this interventional study was conducted in September 2021 before the re-opening of dental schools in Iran during the fifth peak of COVID-19.

The study was based on presenting a designed three-hour webinar for Alborz dental students and assessing their knowledge, attitudes, and practices before and after the intervention using an online developed and validated questionnaire. The inclusion criteria included all dental students who have entered the clinical phase of practice (third year and above), participated in the webinar and completed the pre-and post-intervention questionnaire. Preclinical students who were not practicing dentistry (the first two years) were excluded from the study. Among 360 dental students at the time of the survey in Alborz dental school, 185 clinical students were eligible to participate in the study.

The aims and all study components (intervention and pre-post surveys) were fully explained to the student by sharing an e-poster on their social media groups two weeks ahead as an invitation procedure.

### Educational intervention

In this study, educational content was designed regarding COVID-19 management in dentistry. The aim of preparing this comprehensive content was to improve the knowledge, attitudes, and practices toward this infectious disease among students just before the official re-opening of dental schools in Iran. This content was mainly based on clinical tips selected and collected by the health ministry of Iran, which had been sent to dental schools to educate students during the COVID-19 pandemic, and the latest guidelines and published articles. The final version of the content was reviewed and edited by three dental specialists and consisted of three main aspects. The first part included virology and pathology of coronavirus, signs and symptoms, duration of infection, period of being a carrier after virus exposure, effective masks, seal check, mask disinfection methods in emergencies, virus durability on surfaces, and efficient mouth rinses against the virus. The second part concerned transmission routes, measures to minimize the possibility of producing infectious aerosols, and transmission, correct handwashing method, referral principles of suspected patients, and proper donning and doffing of personal protective equipment (PPE). The last part included patient triage and dental emergencies during the pandemic. In addition, several short educational videos were used to show the correct way of mask-wearing, mask sealing, and proper PPE. Three dental faculties presented this content in a three-hour webinar via Sky room, the national online meeting platform. The presentation was communicative, and participants could ask questions at the end of each section and during break times.

### The questionnaire

In this study, a questionnaire was designed based on the educational content prepared to present as the study intervention. The primary version of the questionnaire, including 52 questions, was validated by eight content experts and pilot tested for reliability on 17 dental students before dissemination. After editing and removing 15 questions according to the experts’ comments, the final version of the questionnaire was prepared in four sections, including (1) Demographic information (age, gender, school semester, self and family history of the disease (parents or siblings living together) (2) Knowledge part, measured by 12 questions regarding pathology, symptoms, diagnosis, preventive measures, and screening in dentistry (3) The attitude part was measured by 10 questions concerning students’ perception of mortality and the necessity of preventive measures (4) evaluating students’ practices in the school dental clinic by 15 questions (PPE, handwashing, appliance disinfection, mouth rinses, and social distancing. Final Cronbach’s alpha for the questionnaire reliability was estimated as 0.7. Knowledge was measured by three choices: true, false, and I don’t know. The correct answer was assigned 1 point, and the incorrect or unknown answer (I don’t know) was given no point. For attitude questions, a five-point Likert scale ranged from 1 = completely disagree, to 5 = completely agree. Moreover, practice questions had a four-point Likert scale ranging from 1 = never to 4 = always. (See the questionnaire in the supplementary material 1)

The online questionnaire was distributed 3 times through Porsline, an electronic survey platform, once a week before the webinar date, the second time, right after the webinar, and third, two months later, as a follow-up survey.

### Data analysis

Descriptive statistics were used for describing the participants’ demographic characteristics (frequency, mean and standard deviation) The repeated measures ANOVA was conducted to assess the difference between the 3 times interventional results (pre, post, and follow-up findings) In addition, Fisher exact test and Chi square, were used to analyze qualitative variables. All statistical analyses were performed using SPSS software version 25. P-values lower than 0.05 were considered to be statistically significant.

### Ethical considerations

The study protocol was approved by the Alborz University of Medical Sciences Ethics committee (number:1400.087), and all the methods were carried out following relevant guidelines. A brief introduction provided information on the aims and objectives of the study, procedure, voluntary nature of participation, and declaration of anonymity and confidentiality were fully explained to students right before the webinar initiation. The participants confirmed their willingness to voluntarily participate in the study by informed consent obtained in the first part of the questionnaire. Students’ id numbers recognized the completed questionnaires, and their names and related information remained confidential.

## Results

### Demographic characteristics

**Fig. 1:**
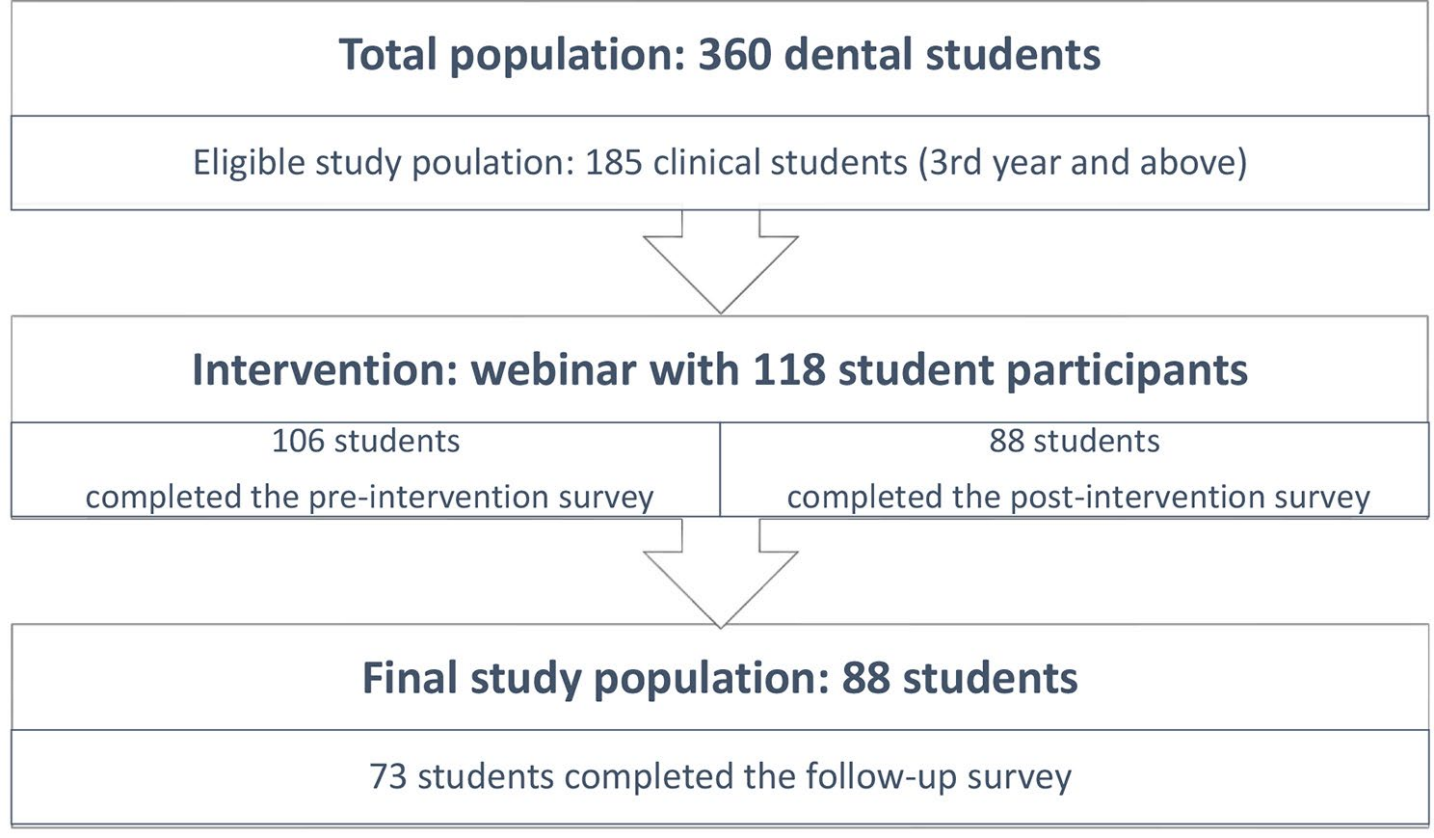
Study sampling and participants recruitments

Among 360 dental students, 185 clinical students were eligible to be included in the study. According to Figure 1, 118 students participated in the webinar, and 88 responded to pre- and post-intervention questionnaires. Out of 88 students, 73 of them completed the follow-up survey.

The frequency and percentage of the demographic characteristics, including gender, semester, and the history of COVID-19 infection in students and their families, are presented in Table 1. The participants were between the fifth and 12th school semesters, participating in clinical dentistry fields. COVID-19 had not infected 65.90% of the participants, and 55.70% had not reported a history of COVID-19 infection in at least one family member. Of the 88 participants, 54.54% were male, with a mean age of 25 ranging from 21 to 40, while the rest were female (45.45%) with a mean age of 24.2 ranging from 21 to 32.

**Table 1:** Demographic characteristics of the participants

Characteristics	Frequency	Percentage (%)
**Gender**		
Male	48	54.54
Female	40	45.45
**School Semester**		
5th	3	3.40
6th	43	48.90
8th	22	25
10th	15	17
12th	5	5.70
**Self-Infection History**		
Positive	30	34.10
Negative	58	65.90
**Family Infection History**		
Positive	39	44.30
Negative	49	55.70
**Total**	88	100

### Knowledge, attitude, and practice scores

Table 2 indicates the significant increase in mean scores of knowledge, attitude, and practice of dental students after the educational intervention and a two-month follow-up survey. The level of knowledge was enhanced more greatly (48.58%) than the attitude (6.37%) and practice (17%) of the students after this educational webinar. The associations between pre-intervention knowledge, attitude and practice scores, and demographic characteristics were statistically insignificant.

**Table 2:** Participants’ knowledge, attitude, and practice scores

	Surveys	Mean ± SD	Effect size	P-value
Knowledge	Pre-intervention	7.78 ± 1.58	0.798	< 0.001*
	Post-intervention	10.66 ± 0.89		
	2-month follow-up	11.56 ± 0.57		
Attitude	Pre-intervention	43.13 ± 3.86	0.317	< 0.05*
	Post-intervention	45.28 ± 2.39		
	2-month follow-up	45.88 ± 1.31		
Practice	Pre-intervention	50.75 ± 5.79	0.728	< 0.001*
	Post-intervention	56.81 ± 3.19		
	2-month follow-up	59.40 ± 0.93		

*: *p* ≤ 0.05(level of significance:0.05), Repeated measures ANOVA

## Discussion

Knowledge regarding COVID-19 is essential for taking preventive measures against its infection [19]. Considering the high risk of COVID-19 exposure in dental clinics due to aerosol-producing procedures, educational interventions are needed to prevent the spread of the virus properly [18, 22]. A previous cross-sectional study indicated the need for educating Alborz dental students about COVID-19 [21]. This study conducted an educational intervention regarding COVID-19, and knowledge, attitude, and practice were assessed before and after the intervention right before dental school re-opening. Schools and universities in Iran had been closed, practicing social distancing and online learning for almost two semesters because of the detrimental impacts of COVID-19 pandemic.

Based on the study findings, all three scores improved after the educational intervention. The knowledge enhanced after the educational webinar; this result is supported by similar previous studies [11, 12]. Kaim and colleagues identified an increase in the perceived knowledge, safety, and individual resilience in an Israeli population through a brief educational video [12]. Moreover, in line with our findings, Ayed and colleagues designed and presented their educational intervention via Whats App groups for secondary students in Egypt and found positive results [11]. Previous study results showed that E-health literacy was significantly correlated with improved knowledge and health behavior [16, 23].

Our results showed dental students’ attitude scores increased slightly after the intervention. However, the slight improvement in attitude (6.37%) shows that greater changes toward COVID-19 might need long-term education and appropriate information presentation strategies. Previously, a study results showed a significant association between dental students’ semester and their attitude toward COVID-19, but we did not find a significant relationship [14] .

Regarding the changes in practice scores, it seems that COVID-19 protective measures in students’ dental practices had improved after the intervention during the school re-opening and in a 2-month follow-up. Moreover, the protocols were sent to students’ social media groups and printed as posters in clinical departments to encourage safe practices.

To the best of our knowledge, this study was the first educational intervention designed for dental students in Iran. It was conducted when most dental students had received two doses of vaccine, and the pandemic was no longer an unknown topic. Therefore, an educational intervention conducted at the beginning of the outbreak could have had a more significant impact on the attitude and practice of the students. The results of this study can help to make changes in related courses such as infection control in the dental curriculum so that students can benefit from well-designed and appropriate programs on different aspects of the upcoming pandemic. Our findings suggest that targeted health educational interventions should be directed to dental students and all dental team members, including faculties, assistants, hygienists, and nurses. Besides, non-medical students could be other targeted populations to build positive knowledge and attitudes and guarantee students’ safety [20].

The major limitation of the present study is that the sample size was restricted to students in one dental school in Iran. Hence, the results could not be generalized to all dental student populations, although findings can encourage educational institutions and other policymakers to cooperate and take appropriate actions. Another limitation is that not all the participants took part in the 2-month follow-up survey, and 15 students were lost in this part of the study.

Future studies could be conducted for all dental team members and non-medical students to investigate the longitudinal impacts of such educational interventions on changing behavioral practices in various aspects of the COVID-19 pandemic.

## Conclusion

In light of the study findings, this first-designed educational intervention significantly improved dental students’ knowledge, attitude, and practice in Iran. Furthermore, the stated research hypothesis was statistically supported. Therefore, it seems that policymakers should focus on creating the well-planned educational intervention programs and training strategies to promote the knowledge, attitude, and practice of all healthcare providers in future crises in the medical field.

## Electronic supplementary material

Below is the link to the electronic supplementary material.

Supplementary Material 1 Questionnaire.

## Data Availability

The datasets analyzed during the current study are available from the corresponding author upon reasonable request.
