# Supplementary material for: Impact of educational intervention regarding COVID-19 on knowledge, attitude, and practice of students before dental school re-opening
Source: BMC Oral Health. 2023 Mar 18;23:156. doi: 10.1186/s12903-023-02845-y (PMC10024005; doi:10.1186/s12903-023-02845-y)
Supplement: Supplementary file 1 — Supplementary Material 1 Questionnaire. [file 12903_2023_2845_MOESM1_ESM.docx]

|  | **Knowledge Questions** | **True** | **False** | **Don’t know** |
| --- | --- | --- | --- | --- |
| 1 | The symptoms of COVID-19 have a sudden onset. |  |  |  |
| 2 | Gastrointestinal symptoms such as abdominal pain, nausea, and vomiting can be symptoms of this disease. |  |  |  |
| 3 | RT-PCR test for coronavirus shows the ability of this disease to spread. |  |  |  |
| 4 | The presence of the virus receptor on the surface of the upper respiratory cells causes the loss of the sense of smell in some affected patients. |  |  |  |
| 5 | Depending on the severity of the symptoms, people with this disease are carriers of the virus for at least 14 days after the symptoms disappear. |  |  |  |
| 6 | Adults are at higher risk than children for COVID-19. |  |  |  |
| 7 | In preventing COVID-19, the correct use of a mask and the right seal is more important than the type of mask. |  |  |  |
| 8 | The virus transmission factor in working with a dental turbine is the aerosols created in the workspace, which can remain in the air for longer. |  |  |  |
| 9 | In the conditions of the COVID-19 pandemic, the first stage of admission (triage) of patients is screening. |  |  |  |
| 10 | Mixing sodium hypochlorite with hot water makes it ineffective for disinfecting virus-infected surfaces. |  |  |  |
| 11 | There is no need to disinfect dental casts to prevent the coronavirus transmission. |  |  |  |
| 12 | Oxidative mouthwashes are chosen to prevent the transmission of COVID-19 before starting dental work. |  |  |  |

Table A1: Participants knowledge, attitude and practice questionnaire

|  | **Attitude Questions** | **Completely agree** | **Agree** | **No idea** | **Disagree** | **Completely disagree** |
| --- | --- | --- | --- | --- | --- | --- |
| 1 | In my opinion, COVID-19 is a very deadly and dangerous disease. |  |  |  |  |  |
| 2 | The risk of this disease is lower for people who have been vaccinated. |  |  |  |  |  |
| 3 | The most common way of contracting COVID-19 among dentists is through dental aerosol. |  |  |  |  |  |
| 4 | A patient with a history of being hospitalized due to Corona infection needs a longer quarantine period after discharge and recovery (14 to 21 days). |  |  |  |  |  |
| 5 | Compliance with the dentist and the patient's protocols effectively prevents infection. |  |  |  |  |  |
| 6 | In my opinion, having an underlying disease in dentists puts them at greater risk of infection. |  |  |  |  |  |
| 7 | Knowing new information and the latest news related to COVID-19 is necessary for personnel to manage and comply with protocols. |  |  |  |  |  |
| 8 | Observing health protocols among dental professors and students is as important as between a dentist and a patient. |  |  |  |  |  |
| 9 | Educating the patient through triage and before entering the examination room is as important as educating the dentists. |  |  |  |  |  |
| 10 | Because the carriers are asymptomatic, the most important way to prevent infection is to comply with the protocols fully and assume that the patient is positive in terms of being infected with COVID-19. |  |  |  |  |  |

|  | **Practice Questions** | **Always** | **mostly** | **rarely** | **never** |
| --- | --- | --- | --- | --- | --- |
| 1 | I use a suitable mask during dental work. |  |  |  |  |
| 2 | I use a shield during dental work. |  |  |  |  |
| 3 | I wear scrubs during dental work. |  |  |  |  |
| 4 | During the pandemic, before starting the dental treatment, I screen the patients and take a detailed history of corona infection. |  |  |  |  |
| 5 | Before performing dental treatment for patients, I wash my hands with soap and water for at least 20 seconds. |  |  |  |  |
| 6 | After performing dental treatment for patients, I wash my hands with soap and water for at least 20 seconds. |  |  |  |  |
| 7 | I use hand sanitizer and surface disinfectants containing alcohol in the dental work environment. |  |  |  |  |
| 8 | I disinfect dental trays and casts before sending them to the laboratory |  |  |  |  |
| 9 | I disinfect the trays and dental casts after delivery from the laboratory. |  |  |  |  |
| 10 | Before starting the dental work, I give the patient an oxidative mouthwash. |  |  |  |  |
| 11 | Before using the mask, I check the mask's seal to ensure adequate protection. |  |  |  |  |
| 12 | I do not treat patients with suspected COVID-19 infection. |  |  |  |  |
| 13 | I recommend home quarantine for suspected patients with corona infection for at least 14 days after recovery. |  |  |  |  |
| 14 | In case of typical symptoms of COVID-19, including cough and fever, I will quarantine myself at home. |  |  |  |  |
| 15 | In case of typical symptoms of COVID-19, including cough and fever, I will take a diagnostic test. |  |  |  |  |
